# Genetic Determinants of Inherited Endocrine Tumors: Do They Have a Direct Role in Bone Metabolism Regulation and Osteoporosis?

**DOI:** 10.3390/genes12081286

**Published:** 2021-08-23

**Authors:** Francesca Marini, Francesca Giusti, Teresa Iantomasi, Maria Luisa Brandi

**Affiliations:** 1 Department of Experimental and Clinical Biomedical Sciences, University of Florence, 50139 Florence, Italy; francesca.marini@unifi.it (F.M.); francesca.giusti@unifi.it (F.G.); teresa.iantomasi@unifi.it (T.I.); 2 Fondazione Italiana Ricerca sulle Malattie dell’Osso, Italian Foundation for the Research on Bone Diseases, 50141 Florence, Italy

**Keywords:** inherited endocrine tumors, oncogenes, tumor suppressor genes, bone tissue, bone modeling, bone remodeling, skeletal clinical features

## Abstract

Endocrine tumors are neoplasms originating from specialized hormone-secreting cells. They can develop as sporadic tumors, caused by somatic mutations, or in the context of familial Mendelian inherited diseases. Congenital forms, manifesting as syndromic or non-syndromic diseases, are caused by germinal heterozygote autosomal dominant mutations in oncogenes or tumor suppressor genes. The genetic defect leads to a loss of cell growth control in target endocrine tissues and to tumor development. In addition to the classical cancer manifestations, some affected patients can manifest alterations of bone and mineral metabolism, presenting both as pathognomonic and/or non-specific skeletal clinical features, which can be either secondary complications of endocrine functioning primary tumors and/or a direct consequence of the gene mutation. Here, we specifically review the current knowledge on possible direct roles of the genes that cause inherited endocrine tumors in the regulation of bone modeling and remodeling by exploring functional in vitro and in vivo studies highlighting how some of these genes participate in the regulation of molecular pathways involved in bone and mineral metabolism homeostasis, and by describing the potential direct effects of gene mutations on the development of skeletal and mineral metabolism clinical features in patients.

## 1. Introduction

The specific clinical affections of bone tissue, and/or the deregulation of bone and mineral metabolism, leading to premature bone mass loss and osteoporosis, develop in a percentage of patients affected by inherited endocrine tumors, as primary manifestations of the syndrome and/or as a consequence of hormone-secreting neoplasms, being a cause of morbidity and disability. 

To date, the medical literature has poorly and marginally documented skeletal affections in inherited endocrine tumors, and a comprehensive and detailed review, focused on the roles of the genetic determinants of these diseases in the regulation of bone and mineral metabolism, and, possibly, in the development of skeletal manifestations, is missing. 

This review wants to offer clinicians an overview of the molecular mechanisms underlying bone and mineral metabolism alterations in different syndromic and non-syndromic inherited endocrine tumors, as possible bases for targeted clinical managements of skeletal pathological manifestations in these congenital diseases. 

In this light, we specifically reviewed the current knowledge, derived from in vitro and in vivo studies, about the possible role that some of the genes, whose germline mutations are responsible for inherited endocrine tumors, may have in the regulation of molecular pathways involved in skeletal development and bone and mineral metabolism homeostasis.

## 2. Inherited Endocrine Tumors

The term “endocrine tumors” indicates a heterogeneous group of neoplasms originating from specialized hormone-secreting cells and affecting the endocrine glands. Tumoral cells maintain their hormone-secreting ability (functioning tumors; FTs) in a majority of cases, or, in a small portion of endocrine tumors, they lose the capability of producing hormones (non-functioning tumors; NFTs). 

Endocrine tumors can develop as sporadic cancers caused by somatic mutations or in the context of familial Mendelian inherited diseases. Congenital forms are caused by germinal heterozygote autosomal dominant mutations in oncogenes or tumor suppressor genes inherited from the affected parent, or, in extremely rare cases, developed de novo at the embryo level. The genetic defect results mainly in a loss of cell growth control in target endocrine cells, leading to tumor development.

Inherited endocrine tumors can be non-syndromic (the affected patient develops only a single endocrine tumor during his/her lifetime) or syndromic (the patient develops multiple endocrine and non-endocrine tumors in different organs during his/her lifetime) [1].

In addition to their specific endocrine and non-endocrine tumors, patients affected by some of these genetic diseases can manifest alterations of bone and mineral metabolism, presenting both as pathognomonic skeletal clinical features and/or non-specific bone and mineral metabolism alterations (Table 1), which can be either secondary complications of endocrine functioning primary tumors and/or a direct consequence of the gene mutation. 

## 3. Genetic Determinants of Inherited Endocrine Tumors and Bone

Bone is a highly dynamic tissue, undergoing life-long remodeling, which is the result of a finely regulated balance between osteoclast bone resorption and osteoblast bone formation [11] and which grants adaptation to external forces, the completion of bone correct functions and the maintenance of skeletal and mineral metabolism health, as well as self-repair after fractures.

In some patients affected by inherited endocrine tumors, the endocrine tumor-derived hormone excess is responsible for the disturbance of the normal osteoclast–osteoblast balance and/or the alteration of correct skeletal modeling and bone and mineral metabolism homeostasis, resulting in the secondary development of skeletal alterations, premature bone mass loss, early onset osteoporosis and an increased risk of fragility fractures.

In some inherited endocrine tumors, a direct involvement of the inherited mutated gene in alterations of bone and mineral metabolism, at cellular and molecular levels, is strongly suspected, being primarily responsible for the development of specific pathognomonic primary skeletal abnormalities.

In this section, we will discuss the ascertained molecular roles of the genes whose germline mutations are responsible for inherited endocrine tumors in the regulation of skeletal modeling and remodeling, and/or in the control of mineral metabolism homeostasis.

### 3.1. CDC73 Gene

The *CDC73* (Cell Division Cycle 73) gene encodes the parafibromin, a transcription factor that is a core member of the Polymerase-Associated Factor 1 (PAF1) complex, which regulates gene transcription and histone modifications. 

Droscha et al. [12] investigated the role of parafibromin in osteoblast differentiation and function by generating two murine models bearing a germinal conditional homozygote deletion of the *Cdc73* gene, one in Mesenchymal Stem Cell (MSC) progenitors and the other in mature osteoblasts and osteocytes. Parafibromin was shown to be mandatory for MSC differentiation and survival; the conditional complete loss of *Cdc73* expression in MSCs induced a high-rate apoptosis within the mesenchyme, blocking the correct organ development at embryo day 11.5. Conversely, the mice completely missing *Cdc73* in osteoblasts and osteocytes were viable and lived well beyond one year. Surprisingly, they manifested an overall significant increase in whole-body Bone Mineral Density (BMD). Long bones appeared thicker and stronger. At the microarchitectural level, these bones were characterized by large pores in the cortical bone area, undergoing an active bone remodeling associated with a significantly increased mineral apposition rate. At the same time, the osteocytes in the cortical bone had a high rate of apoptosis. The trabecular bone presented a significant increase in both osteoblast number and osteoid bone volume, coupled with a significantly decreased number of osteoclasts on the bone surface. At transcriptional level, the knockout of the *Cdc73* gene led to a decrease in the expression of osteoblast-specific genes, mainly those encoding collagen and bone matrix proteins. Taken together, these data suggest that parafibromin is necessary for MSC survival and, at the same time, may act as a transcriptional repressor of activity of maturing osteoblasts, having a possible direct role in the regulation of bone-forming cell differentiation and activity, and bone homeostasis.

Ossifying fibromas are benign fibro-osseous neoplasms originating from pluripotent MSC and are capable of forming mineralized bone tissue and cement. Although ossifying fibromas are part of the Hyperparathyroidism-Jaw Tumors (HPT-JT) syndrome, a direct role in the tumor development of either an haploinsufficiency or an aberrant intracellular expression of parafibromin in bone-forming cells has not yet been clearly demonstrated. Recently, Costa-Guda et al. [13] analyzed parafibromin expression and intracellular localization in nine sporadic human non-syndromic ossifying fibromas. Four cases (44.4%) showed a complete absence of nuclear parafibromin expression, one of them presenting an aberrant cytoplasmatic expression of the protein. The full sequence of the *CDC73* gene was available only for three cases, revealing the presence of one homozygote and one heterozygote somatic inactivating mutation of the gene, both cases showing a complete absence of nuclear parafibromin expression. Genomic DNA was not available for the performance of germline mutation screening. 

The data from the above-described studies provided, both in an animal model and in humans, concordant evidence that the loss of the nuclear expression of parafibromin in bone-forming cells may have an important, direct, role in the pathogenesis of ossifying fibromas, confirming these skeletal neoplasms as a primary clinical manifestation and direct consequence of the genetic defect in the *CDC73* gene. 

### 3.2. GCM2 Gene

The *GCM2* (Glial Cells Missing Transcription Factor 2) gene encodes the Glial Cells Missing Homolog 2 (GCM2) protein, a zinc finger-type transcription factor, which is essential for the correct embryonic development of parathyroid glands. Recently, Yamada et al. [14] showed that the reduction in GCM2 expression is responsible for a reduction in parathyroid cell proliferation, an increase in parathyroid cell apoptosis and an attenuation of parathyroid function, demonstrating a key role of this protein in the maintenance, proliferation and activity of adult parathyroids. Parathyroid glands are the principal actors of calcium homeostasis, and subsequently, of bone mineralization. Thus, mutations of the *GCM2* gene, which alter the normal development, maintenance and function of these glands, have a repercussion on the mineralization of the extracellular matrix of bone tissue. In particular, gain-of-function mutations are responsible for parathyroid hyperplasia/adenoma with a constant over-secretion of Parathyroid Hormone (PTH) and hypercalcemia, resulting in increased osteoclast activity, a continuous release of calcium ion from bone tissue and a subsequent reduction in mineralized bone mass, leading to early onset osteopenia and osteoporosis with an enhanced praecox risk of fragility fractures. 

### 3.3. APC Gene

The *APC* (Adenomatous Polyposis Coli) gene encodes the homonymous tumor suppressor protein, which acts as a strong negative regulator of the Wnt signaling pathway by directly binding the key transducer of this pathway, the β-catenin, and promoting its rapid degradation. In the absence of a Wnt signal, cytosolic β-catenin is degraded by the ubiquitination/proteasome system upon its binding and phosphorylation to a multi-protein destruction complex that comprises APC. The binding of Wnt to LRP5 or LRP6 results in the inactivation of the destruction complex and the subsequent accumulation of the cytosolic β-catenin, favoring its nuclear translocation and the transcriptional activation of Wnt target genes that leads to increased cell growth and reduced apoptosis. Mutated APC proteins mimic the Wnt signal, leading to an increase in the cytosolic β-catenin and to the consequent β-catenin-dependent induction of the expression of Wnt target genes. The efficiency of β-catenin downregulation by the destruction complex is compromised in the presence of a mutated APC protein, based on the site of the mutation [15]. The important role of the Wnt cascade in the regulation of osteogenesis and in the pathophysiology of a number of skeletal diseases is now well-established [16,17]. Indeed, increased levels of the canonical Wnt/β-catenin signaling inhibit the expression and activity of SRY-Box Transcription Factor 9 (SOX9) and stimulate the expression of Runt-related Transcription Factor 2 (RUNX2), two commitment factors of chondrocyte and osteoblast differentiation, respectively, decreasing chondrogenesis and promoting osteogenesis [18,19].

Miclea et al. [20] created a conditional knockout mouse model for the *Apc* gene in Col2aI-expressing cells to investigate whether this gene was involved in the lineage commitment of skeletal precursor cells. The homozygote *Apc*-knocked out animals died perinatally due to severely impaired skeletogenesis, affecting both the axial and appendicular bone segments. Analyses on homozygote embryos showed, in the *Apc*-knocked out cells, that the complete absence of the Apc protein resulted in an accumulation of cytoplasmic β-catenin, which, subsequently, translocated into the nucleus; highly elevated levels of β-catenin were found at all the sites of endochondral ossification and within the chondrocyte nucleus. The cells lacked expression of both chondrogenic (i.e., Sox9) and osteogenic markers (i.e., Col2aI), demonstrating that the conditional loss of functional Apc in skeletal precursors inhibited mesenchymal cell condensation and chondrogenic differentiation. Conversely, the conditional heterozygous inactivation of *Apc* did not interfere with embryonic skeletal development, postnatal growth or bone acquisition. Heterozygote *Apc*-knocked out embryos, at various developmental stages, showed a normal spatio-temporal expression of chondrogenic and osteogenic markers, and no detectable levels of β-catenin were found. The micro-computed tomography of the distal femora performed in the heterozygote *Apc*-knocked out mice, monitored for 12 weeks after birth, showed no significant difference compared to the wild type littermate mice in BMD, trabecular bone volume fraction, trabecular number, trabecular thickness and trabecular separation. Microscopical analysis of bones at 24 weeks after birth showed no important anomalies in the skull, ribs, vertebral column and long bones. These data imply that the level of wild type Apc protein produced by a single functional *Apc* allele is sufficient to grant the Apc-mediated appropriate β-catenin degradation and the correct Wnt-regulated skeletal modeling.

The importance of the APC protein for the correct skeletogenesis has also been shown by Holmen et al. [21]. The mice with the conditional homozygote osteoblast-specific deletion of the *Apc* gene manifested growth retardation and dramatic defects in bone development, characterized by severe osteopetrosis associated with disturbances in bone architecture and composition, leading to mortality within two weeks after birth. At bone cell levels, the depletion of the *Apc* gene in osteoblasts, and the subsequent deregulation of β-catenin signaling, caused a cell-autonomous osteoblast defect and, at the same time, altered bone resorption, by dramatically reducing the number of osteoblasts, as a consequence of the decreased expression of Receptor Activator of Nuclear Factor-κ B Ligand (RANKL) and the increased expression of Osteoprotegerin (OPG). 

Although variations in bone mass are not a clinical hallmark of Familial Adenomatous Polyposis (FAP), two studies have shown a direct association between the presence of an *APC* mutation and BMD values in humans [22,23]. A cross-sectional study on 30 FAP patients bearing a germinal heterozygote mutation of the *APC* gene showed a significantly higher mean BMD at the lumbar spine, total hip, femoral neck and trochanter in FAP patients, with respect to age- and gender-matched healthy controls, in the presence of a balanced bone remodeling, as displayed by the mean concentrations of procollagen type I N-terminal propeptide and β-crosslaps being within the normal ranges [22].

Chew et al. [23] showed that individuals bearing a heterozygote deletion encompassing part of the *APC* gene had a higher BMD at the forearm, spine and total hip compared to subjects with two wild type copies of the gene. 

Primary osteomas develop as disease-specific clinical manifestations in a percentage of patients with FAP. These benign bone tumors affect bone segments characterized by intramembranous ossification, an ossification process in which mesenchymal cells differentiate directly into osteoblasts and deposit mineralized bone. *APC* mutations, that are responsible for β-catenin accumulation and mimes the Wnt signaling, may suppress the chondrogenic potential of the mesenchymal osteochondral progenitor cells, favoring osteoblast differentiation and intramembranous ossification, and being, thus, directly responsible for the development of osteomas. 

### 3.4. MEN1 Gene

The *MEN1* (Multiple Endocrine Neoplasia type 1) gene encodes a nuclear scaffold protein named menin, which controls gene expression, cell signaling, cell growth, DNA repair and histone modifications. A direct involvement of menin in the regulation of osteogenesis has been widely demonstrated by in vitro and in vivo studies. 

Wild type menin is required for the commitment, in vitro, of the pluripotential MSCs to the osteoblast lineage. The specific menin inactivation by antisense oligonucleotides, in MSC progenitors, inhibits osteoblastogenesis by blocking the Bone Morphogenetic Protein 2 (BMP2) transcriptional activity of Smad1/5 and, thus, antagonizing Alkaline Phosphatase (ALP) activity and the expression of the key osteoblast transcription factor RUNX2, and of the protein of the bone extracellular matrix-like type I collagen and Osteocalcin (OCN) [24]. The inactivation of menin has no effect on the expression of adipogenic and chondrogenic markers in MSCs induced to differentiate into the adipocyte or the chondrocyte lineage, respectively. Conversely, menin inactivation in the murine osteoblastic cell line MC3T3-E1 has been shown to not affect BMP2-stimulated ALP activity nor the expression of RUNX2 and OCN, confirming the inductive role of menin in the commitment of osteoblast precursors, but not on mature osteoblasts. In addition, the fact that the stable inactivation of menin in MC3T3-E1 cells increased ALP activity, mineralization and the expression of type I collagen and OCN indicates that menin exerts an inhibitory role in osteoblast later differentiation [24]. In normal conditions, menin interacts physically and functionally with BMP2, and with the BMP2 signaling molecules Smads 1/5, in still uncommitted MSCs, inducing their osteoblast differentiation [25]. After the osteogenic commitment, the interaction of menin with BMP2 and Smads 1/5 is lost, in the differentiated osteoblasts, in which menin physically interacts with Smad3, positively regulating the Transforming Growth Factor-β (TGFβ)/Smad3 pathway and negatively regulating the BMP2/Smad1/5- and the RUNX2-induced transcriptional activities and, thus, leading to the inhibition of osteoblast late-stage differentiation [26].

The conditional homozygote knockout mice for the *Men1* gene in mature osteoblasts showed significantly reduced BMD compared to the wild type animals [27]. Bone mass reduction affected both trabecular and cortical bone, with significantly reduced trabecular bone volume and number, associated with an abnormal structure of trabeculae, and significantly reduced cortical bone volume, surface and thickness. At the cellular levels, the mutated mice had significantly reduced osteoblast and osteoclast numbers and increased osteocyte numbers, coupled with a reduced mineral apposition rate. The isolated osteoblasts from the knockout mice showed a reduced expression of osteoblast markers, together with an increased expression of both osteocyte markers and pro-apoptotic genes. Conversely, a transgenic mouse model overexpressing menin in osteoblasts manifested a gain of bone mass, increased bone volume and trabecular numbers, increased numbers of mature and active osteoblasts, increased osteoblast differentiation markers (Runx2, OCN and type I collagen) and an enhanced ALP activity and mineral apposition rate.

Liu et al. [28] confirmed that the conditional homozygote depletion of *Men1* in the osteoblast lineage of mice caused osteoporosis. The *Men1* deficiency specifically induced in osteocytes, but not in early differentiated osteoblasts, enhanced osteoclastogenesis (increased osteoclast numbers and surface) and bone porosity (reduction in the number of trabeculae and increased trabecular separation) in vitro and in vivo. Osteocytes lacking *Men1* showed an enhanced secretion of C-X-C Motif Chemokine 10 (CXCL10), a known osteoclastogenesis-inducing factor.

Luzi et al. [29] showed that menin induces, in human MSCs induced in vitro to differentiated into osteoblast lineage, the expression of the miR-26a, an osteogenesis-inhibitor microRNA that negatively regulates the expression of SMAD1 during osteoblast differentiation of the MSCs [30], acting as a transcription factor and directly occupying the miR-26a gene promoter. These data indicate the miR-26a-SMAD1 as an additional regulative pathway by which menin inhibits the osteoblast late-stage differentiation of committed precursors.

Patients affected by MEN1 syndrome do not develop any primary bone affections, directly associated and derived by *MEN1* mutations. MEN1 patients have a higher risk of early onset osteoporosis, with respect to the general population of the same age [5], attributed to primary hyperparathyroidism (PHPT) and other endocrine dysfunctions typical of the syndrome. A direct role of *MEN1* gene mutations in the premature bone mass loss has not been demonstrated; however, given the fact that menin protein has been shown to be directly involved in the regulation of osteoblastogenesis, a synergical pro-osteoporotic effect of menin haploinsufficiency in mesenchymal stem cells and bone forming cells cannot be excluded.

### 3.5. RET Gene

The *RET* (REarranged during Transfection) gene encodes the homonym tyrosine kinase transmembrane receptor, RET. Binding of the ligands stimulates RET receptor dimerization and the activation of downstream signaling pathways, which play a role in cell survival, proliferation, differentiation and migration.

Patients affected by Multiple Endocrine Neoplasia type 2B (MEN2B) develop typical skeletal abnormalities, not manifesting in the Multiple Endocrine Neoplasia type 2A (MEN2A) form.

The murine embryonal fibroblast NIH 3T3 cell line expressing RET-MEN2B mutants or stimulated with the Glial Cell Line-Derived Neurotrophic Factor (GDNF), a biological ligand of the RET receptor, showed a highly induced expression of Stanniocalcin 1 (STC1) [31], a protein involved in early skeletal development and joint formation [32]. Conversely, the NIH 3T3 expressing RET-MEN2A mutants had no effect on the expression of STC1, suggesting that the *STC1* gene expression may have a role in the development of MEN2B-specific bone phenotype.

A comparison of the gene expression profile between the specimens of Medullary Thyroid Carcinoma (MTC) from MEN2A and MEN2B patients showed that the malignant C cells of MEN2B MTCs expressed high levels of Chondromodulin 1 (CHM1) [33], a secreted glycoprotein known to be a regulator of cartilage and bone growth that is normally expressed in proliferating chondrocytes of the growth plates. Interestingly, all the MEN2B MTC patients with skeletal abnormalities had high CHM1 expression, suggesting that the CHM1 aberrant expression in the MEN2B MTC, in early infancy and childhood, could be responsible for alterations at the growth plates of developing bones, leading to the skeletal abnormalities that characterize the MEN2B form of the syndrome. 

In addition, GDNF, one of the major ligands of the RET receptor, has been shown in vitro to induce the migration of Bone Marrow MSCs (BMMSCs) and stimulate osteogenesis [34]. The pre-osteoclast conditioned medium was demonstrated to contain GDNF that bound GFRα1 and recruited the co-receptor RET, inducing RET phosphorylation in BMMSCs, resulting in cell migration and differentiation toward the osteoblast lineage. Inhibition of the GDNF/GFRα1/RET signaling pathway blocked both BMMMSC migration and osteogenic differentiation. These data indicate the existence of GDNF-mediated osteoclast-osteoblast crosstalk, which leads to BMMSC migration and osteogenic differentiation via the activation of RET tyrosine kinase activity. Mutant RET receptors, constitutively activated for their tyrosine kinase activity, could mimic this GDNF/GFRα1/RET signaling, being responsible for the development of bone manifestations in MEN2B.

Multiple Endocrine Neoplasia type 2 (MEN2) is characterized by a strict genotype-phenotype correlation, not only limited to endocrine tumor development but also to the displaying of pathognomonic skeletal abnormalities that manifest only in patients with the MEN2B form. The *RET* mutations causing MEN2A affect the extracellular domain of the receptor miming a constitutive binding with the ligand, while the mutations causing MEN2B are located in the two intracellular tyrosine kinase domains of the receptor, resulting in a constitutively activated tyrosine kinase activity; only the latter mutations are responsible for the development of typical skeletal features, presumably via the specific induction of the overexpression of STC1 and CHM1 proteins. Conversely to all the other causative genes of hereditary endocrine tumors, which are tumor suppressor genes, *RET* is a proto-oncogene, and a monoallelic activating mutation is sufficient to alter the function of the receptor and lead to the development of the clinical phenotype, including skeletal abnormalities.

### 3.6. CDKN1B Gene

The *CDKN1B* (Cyclin Dependent Kinase Inhibitor 1B) gene encodes the p27^kip1^ cyclin-dependent kinase inhibitor that binds to and prevents the activation of cyclin E-CDK2 and cyclin D-CDK4 complexes, negatively regulating the cell cycle progression at G1. Some in vitro and in vivo studies have indicated a possible involvement of p27^kip1^ in the regulation of osteoblast differentiation from mesenchymal precursors.

The in vitro culture of bone marrow-derived progenitors, established from mice with the homozygote deletion of the *Cdkn1b* gene, showed that the complete ablation of p27^kip^ resulted in a precocious proliferation of these cells and in an acceleration of their osteoblast differentiation, with respect to those of wild type littermate animals [35]. In fact, when the p27 ^kip^-lacking bone progenitor cells were induced to differentiate into osteoblasts, they showed a higher ALP positive staining, an increased number and size of the osteogenic colonies, and an earlier mineralization of the extracellular matrix, compared with cultures from the wild type mice. In the proliferating osteoblast precursors from the wild type mice, the levels of p27^kip1^ were low, and they substantially increased during osteogenic differentiation. The p27 ^kip1^ null mice showed an increased size of long bone and an increased width of diaphyseal cortical bone shafts compared to wild type littermates, with both the osteoblasts and osteoclasts on trabecular bone surfaces of a normal appearance and a greater cellular density within the bone marrow. 

Zhu et al. [36] showed that the homozygote p27^kip^-null mice (p27^−/−^ mice) presented an increased length of long bones, size of epiphyses, BMD, trabecular bone volume, number of mature osteoblasts and of chondrocytes positive for the proliferating cell nuclear antigen staining, as well as ALP-, type I collagen- and OCN-positive bone areas. When a compound null mouse model bearing both the biallelic deletion of the *Cdkn1b* gene and the homozygote mutation of the parathyroid hormone-related peptide (Pthrp) KI (p27^−/−^Pthrp KI mice) was analyzed, it showed all the above listed bone and cartilage paraments being reduced with respect to the wild type mice but increased with respect to the Pthrp KI mice. These data suggest that the complete depletion of p27^kip^ in the homozygote Pthrp KI mice can partially rescue defects in skeletal growth and osteoblastic bone formation by enhancing endochondral bone formation and osteogenesis. Given the fact that p27^kip^ directly interacts with the C-terminal region of PTHrP, it is conceivable that the p27^kip^ pathway may act downstream to PTHrP in the regulation of skeletal growth and development.

To date, very few clinical cases of Multiple Endocrine Neoplasia type 4 (MEN4) have been reported worldwide, and targeted studies on bone complications in this syndrome are totally missing. As for MEN1, of which MEN4 is a clinical phenocopy, the prematurely manifesting reduced bone mass is mainly attributed to PHPT, and it is thought to manifest later in life and in a less severe degree, with respect to MEN1 patients, accordingly to a later onset and milder PHPT that characterizes MEN4. A direct role of the p27 ^kip1^ haploinsufficiency in altering bone metabolism in MEN4 patients remains to be demonstrated. 

### 3.7. PRKAR1A Gene 

The *PRKAR1A* (Protein Kinase CAMP-Dependent Type I Regulatory Subunit α) gene encodes the type 1A regulatory subunit of the tetrameric cAMP-dependent Protein Kinase A (PKA). While the inactivating mutations in the *PRKAR1A* gene are responsible for Carney Complex (CNC), the gain-of-function mutations of this gene have been identified in patients with acrodysostosis, a heterogeneous group of rare skeletal dysplasia [37,38], suggesting the possibility of a direct involvement of the PKA-regulated pathway in regulating bone modeling and/or remodeling, and presumably being responsible for the development of both chondrodysplasia and osteochondromyxomas when deregulated. 

The heterozygote *Prkar1a* activating point mutation R368X knock-in mice (*Prkar1a* ^[*R368X*]/[+]^ mice) had chondrodysplasia and peripheral acrodysostosis affecting the endochondral skeleton, which resulted in shorter tails, shorter length and shorter forelimbs and hindlimbs, compared to their wild type littermates. Brachydactyly and snout dysostosis were also present, and BMD and bone surface were both significantly reduced [38]. The mutant mice presented a striking delay in the mineralization of the cartilage and epiphyseal secondary ossification centers of forelimb and hindlimb bones, accompanied by modifications in the proliferative and hypertrophic chondrocyte layers. The data from this study showed the importance of the Prkar1a subunit of PKA in the correct regulation of endochondral ossification; however, no defect in the endochondral bone development (i.e., no increased proliferation or decreased chondrocyte differentiation) has been described in human CNC patients.

Conversely, the heterozygote knockout mice for the *Prkar1a* gene (*Prkar1a^+/−^* mice), which mimic the Prkar1a subunit haploinsufficiency of the CNC, developed, in about 80% of cases, tumoral lesions on the tail vertebrae and the sacroiliac region, which arise from cells of the osteoblast lineages [39] and present a histological and cytological similarity to the osteochondromyxomas that develop in human CNC patients. As previously demonstrated in bone tumors arising in human patients with a *PRKAR1A* mutation [40], tumoral osteoblasts from the *Prkar1a^+/−^* mice showed a significantly elevated level of both a total and a free PKA enzyme. 

Osteochondromyxomas are primary, extremely rare, benign bone tumors, manifesting prevalently within the CNC syndrome, composed by a mix of chondroid and osteoid matrix. Data from the study of Pavel et al. [39] indicated that this bone tumorigenesis is predominantly driven by an excessive activation of PKA signaling, as a direct consequence to a *PRKAR1A* inactivating mutation.

### 3.8. PTEN Gene

The *PTEN* (Phosphatase and TENsin Homolog) gene encodes a phosphatidylinositol-3,4,5-trisphosphate 3-phosphatase that negatively regulates the PI3K/AKT/mTOR signaling pathway and cell survival, proliferation and migration. The PTEN/PI3K/AKT axis has been involved in embryonic bone development and fracture healing. 

The conditional knockout of the *Pten* gene in osteoblasts of a mouse model resulted in an increased fracture healing, with a larger and more ossified callus and an improvement of both the early intramembranous bone formation and the later endochondral bone formation during the healing process [41], indicating that the inhibition of PTEN may have a clinical application for enhancing fracture healing in cases of bone non-unions.

The mice with the homozygote conditional deletion of the *Pten* gene in osteoblasts showed a significantly increased whole-body BMD, compared to wild type littermates, that progressively increased throughout life [42]. The mutant mice had a pronounced higher bone volume, both at the cortical and trabecular bone, with an increased cortical thickness in the femur and with the trabeculae being thicker and less separated, than those of the controls. These data suggest that the loss of *Pten* could influence bone formation via intramembranous ossification. At bone cell levels, in vitro cultures of *Pten*-deficient osteoblasts showed a constitutive activation of Akt signaling and target genes and a reduction in the apoptosis rate, with respect to wild type control osteoblasts. 

The mice with the conditional homozygote deletion of the *Pten* gene in osteo-chondroprogenitor cells showed, as did mouse mutants on the osteoblasts, a bone overgrowth, and confirmed the development of an increased skeletal diameter, increased cortical thickness and enhanced number of trabeculae [43]. The chondrocytes, within bone growth plates, appeared disorganized, showing, compared to the control mice, a less well-defined demarcation between the proliferative zone and the beginning of the prehypertrophic region. The primary spongiosa exhibited increased numbers and sizes of disorganized trabeculae, and the nascent perichondrial bone collar showed an accelerated hypertrophic osteoblast differentiation and an enhanced apposition of matrix proteins.

Taken together, these two studies show that PTEN is an important modulator in determining the extent of normal cartilage and bone development and, thus, skeletal size. The PTEN function in chondrocytes is essential for inhibiting dyschondroplasia. As a consequence, the loss of PTEN protein may have a direct pathogenic role in the skeletal manifestations observed in patients with the Cowden/PTEN hamartoma tumor syndrome.

Bone morphogenetic protein 9 (BMP9) is one of the most efficacious osteogenic cytokines. PTEN has been demonstrated, in vitro, to be downregulated by BMP9 during the osteoblast differentiation of mouse embryonic fibroblasts [44]. Some years later, Li et al. [45] demonstrated the existence of a reciprocal feedback between PTEN and BMP9, showing a direct inhibitory action of PTEN on the osteogenic potential of BMP9, which resulted in the inhibition of PI3K/AKT signaling. In vitro analyses showed that PTEN reduced the BMP9-induced osteogenic differentiation of murine C3H10T1/2 MSCs by inhibiting both the mRNA and protein levels of Runx2 and OCN, and by inhibiting ALP activity and mineralization. During osteoblastogenesis, the Wnt10b, a secreted protein member of the canonical Wnt signaling, is upregulated by BMP9, acting as a bone formation promoter and a mediator of the osteogenic potential of BMP9, and favoring the differentiation toward the osteogenic lineage at the expense of adipogenesis. In the C3H10T1/2 cell line, Wnt10b was demonstrated to be able to partially reverse the inhibitory effect of PTEN on BMP9-driven osteogenesis, suggesting that PTEN may inhibit the osteogenic potential of BMP9 by negatively regulating Wnt10b expression, with a mechanism that is still unclear [45]. 

A similar inverse association was also demonstrated between PTEN and BMP2 [46] in the C3H10T1/2 cell line. PTEN inhibited the BMP2-induced osteoblast differentiation of MSCs at the osteoblast lineage commitment level and at the early and late stages of osteogenic differentiation, while BMP2 repressed PTEN expression, leading to the activation of PI3K/AKT and to osteogenesis. PTEN was shown to inhibit the proliferation, but promote apoptosis, of the C3H10T1/2 MSCs. The authors studied the role of the PTEN/PI3K/AKT signaling pathway in ectopic calcification by establishing a specific animal model via the implantation of human recombinant BMP2 into the muscle tissue of mice to induce soft tissue ossification. The inactivation of the PI3/AKT signaling, by PTEN, markedly suppressed the BMP2-induced ectopic endochondral calcification of muscles [46].

Both these studies supported the notion that the activation of the PI3K/AKT signaling pathway is a key step for both BMP2- and BMP9-induced osteoblastogenesis, bone formation and endochondral ossification. Therefore, the PTEN/PI3K/AKT axis represents a potential target for either favoring endochondral fracture repair or for the molecular prevention/therapy of the heterotopic ossification of soft tissues. 

### 3.9. NF1 Gene

The *NF1* (Neurofibromin 1) gene encodes neurofibromin 1, a negative regulator of the Ras signal transduction pathway.

Several studies have documented a decreased BMD in individuals with neurofibromatosis type 1 (NF1) [47,48,49,50,51,52], suggesting that the NF1 haploinsufficiency may contribute to abnormal bone remodeling, either directly or indirectly by aberrant Ras signaling, potentially predisposing NF1-affected individuals to skeletal abnormalities and osteoporosis. Pathognomonic bone manifestations in NF1 syndrome are congenital and primary dysplasias, implying a direct role of an *NF1* defect in the alterations of both skeletal development and bone homeostasis. The MSCs derived from Nf1^+/−^ mice showed an impaired osteoblast differentiation, presumably as a direct consequence of Nf1 protein haploinsufficiency [53]. However, the somatic loss of heterozygosity at the *NF1* locus, found in bone cells from dysplasia and pseudoarthrosis of the tibia in NF1 patients, suggests that the biallelic loss of *NF1* function is likely to be required to cause the bone phenotype in human NF1 syndrome, and confirms that the neurofibromin-Ras signal transduction pathway is directly involved in tibial bone dysplasia [54]. 

A transcriptome profiling of bone cells, derived from a tibial pseudoarthrosis of a NF1 patient, showed that *EREG* and *EGFR1* genes, encoding, respectively, epiregulin and its receptor Epidermal Growth Factor Receptor 1, were among the *NF1*-deficiency-associated top up-regulated genes [55]. An over-expression of the epiregulin gene and protein was shown in human bone cells bearing *NF1* double hit mutations [56]. A three-time over-expression of epiregulin was also confirmed in *Nf1*-deficient mouse bone marrow stromal cells, with respect to wild type controls, both at the mRNA and protein levels, showing that this molecular signature is conserved among mice and humans, and it may have a direct role in the bone abnormalities of the NF1 syndrome. 

The study of the role of *NF1* inactivating mutations in bone modeling and remodeling in vivo is difficult since the homozygote Nf1^−/−^ mice die in utero before bone formation can be studied, and heterozygote Nf1^+/−^ mice have been shown to not manifest any bone phenotypes. 

Kuorilehto et al. [57] studied *Nf1* gene and protein expression profiles in bone tissues from Nf1^+/−^ and wild type mice at 12.5–15.5 embryonic days and in adulthood. Nf1 mRNA and protein were both high expressed in resting, maturation and hypertrophic chondrocytes at the growth plates. The expression patterns of *Nf1* mRNA and protein in the growth plate cartilages of femora and tibias of adult mice were similar to those observed in the embryonic counterparts. The synovial cartilage also resulted as positive for *Nf1* mRNA and protein expression. The osteoclasts, osteoblasts and osteocytes in the periosteum of the adult animals all appeared strongly positive for neurofibromin protein expression. No differences were found for neurofibromin expression between the mutant and wild type animals, both at embryo levels and adulthood, being consistent with the absence of skeletal abnormalities in the heterozygote *Nf1* mutant mice. A neurofibromin-mirroring negative labeling for the phosphorylated p44/42 MAPK complex, as the result of the Ras pathway activation and the consequent p42 and p44 MAPK phosphorylation, was found in proliferative, maturing and synovial cartilages of both the *Nf1*-deficient and wild type skeletal tissues from the embryonic and adult mice. The hyperthropic chondrocytes of the Nf1^+/−^ embryos presented a high expression of phosphorylated p44/42 MAPK, suggesting that the activation of the Ras-MAPK pathway, resulting from NF1 protein haploinsufficiency, may be involved in the development of congenital skeletal dysplasias in NF1 patients.

Kolanczyk et al. [58] generated a mouse model bearing the heterozygote conditional inactivation of the *Nf1* gene in undifferentiated MSCs of the developing limb skeleton (Nf1^Prx1^ mice). Similar to NF1-affected patients, the Nf1^Prx1^ mice showed retarded and reduced bone growth and abnormal growth plates and manifested the anterolateral bowing of the tibia that started in the uterus, was already present at birth, and progressively worsened after two weeks of age. The affected bones were characterized by a decreased calcium content and the increased porosity of the cortical areas. The in vitro analysis of osteoblast precursors extracted from Nf1^Prx1^ mice, and induced to differentiate into osteoblasts, showed a significant reduction in both ALP activity and extracellular matrix mineralization, with respect to those from wild type littermates. Mutant osteoblasts showed a strong induction of osteopontin, a marker of the early differentiation stage and a known inhibitor of calcification, and a reduction in the expression of integrin-binding sialoprotein and OCN, two markers of the terminally differentiated mature osteoblasts. Therefore, the loss of *Nf1* function in osteoblast precursors resulted in an accumulation of incompletely differentiated osteoblasts and, ultimately, in abnormal bone formation. In addition, the Nf1^Prx1^ mice manifested cartilaginous fusions of the hip joints in all the mutated animals, varying from partial to complete fusions, a phenotype that is not observed in NF1 syndrome. The joints from the wild type animals showed a normal nuclear distribution of Sox9, an essential regulator of chondrocyte differentiation from precursors, at embryonic day 13.5, with a shift of Sox9 localization to the cytoplasm at 14.5 embryonic days, which reduced chondrocyte activity and favored correct bone growth. Conversely, the Sox9 nuclear localization persisted in the cartilage joint cells from the Nf1^Prx1^ mice, leading them to remain active chondrocytes, concurring with the joint fusion [58].

The data from the studies described above confirmed NF1 as an important modulator of skeletal development and growth; somatic loss of the wild type protein in osteoprogenitor cells is presumably a key step in the development of NF1-associated bone phenotypes.

### 3.10. TSC1 and TSC2 Genes

The *TSC1* (Tuberous Sclerosis 1) gene encodes for hamartin, a protein that plays a key role in the regulation of cell adhesion, while the *TSC2* (Tuberous Sclerosis 2) gene encodes for tuberin, a GTPase-activating protein that regulates the GTP binding and hydrolyzing activity of the Ras protein superfamily, helping to regulate cell growth, proliferation and differentiation. These two proteins bind to each other, via their respective coiled-coil domains, to form an intracellular hamartin-tuberin complex that negatively regulates the Mammalian Target of Rapamycin Complex 1 (mTORC1) signaling, which is a major regulator of anabolic cell growth. Disruption of the hamartin-tuberin complex, through the loss (inactivating mutations) of either the *TSC1* or *TSC2* gene, results in a constitutive activation of mTORC1.

In recent decades, few studies have investigated the role of mTORC1 signaling in bone cells. Unfortunately, homozygote knockout mice, either for *Tsc1* or *Tsc2* genes, present an early embryonic lethality that prevents the possibility to study the molecular mechanisms by which the dysregulated mTORC1 signaling affects postnatal skeletal development and bone metabolism. 

Fang et al. [59] generated a conditional knockout mouse model selectively lacking both copies of the *Tsc1* gene in Neural Crest-Derived Cells (NCDCs), which normally contribute to the formation of a majority of craniofacial bones. The mutated animals manifested a spectrum of sclerotic craniofacial bone lesions (thickened frontal calvaria, zygomatic bones and nasal bones), comparable to the patients affected by Tuberous Sclerosis Complex (TSC); no increase in the parietal bones of mesodermic origin was found. The *Tsc1* deletion-caused mTORC1 hyperactivation in NCDCs led to an enhanced proliferation of the osteoprogenitor pool at an early postnatal stage, associated with an increased bone formation rate and elevated mineral apposition rate. No effect of mTORC1 signaling was exerted on the number and activity of osteoclasts. The mouse heterozygote for the conditional *Tsc1* deletion had a similar bone thickness to the wild type littermates, indicating that the *Tsc1* haploinsufficiency alone does not affect the osteogenic differentiation of NCDCs. 

When the conditional homozygote deletion of the *Tsc1* gene is induced in Osterix-positive cells, mutant mice show a significantly decreased trabecular bone mass, associated with contemporary reduced osteoblastogenesis and enhanced osteoclastogenesis, and with increased adiposity of the bone marrow [60]. At the bone cell level, hamartin-deficient murine bone marrow stromal cells showed decreased proliferation, reduced early and terminal osteoblast differentiation and increased adipogenic differentiation. Main master osteoblastic transcription factors (Runx2 and Osterix) and osteoblast differentiation markers (ALP, Col1a1 and bone sialoprotein) all showed a decreased mRNA expression, while the expression of master adipogenic transcription factors resulted increased (i.e., peroxisome proliferator-activated receptor γ, fatty acid binding protein 4, adiponectin, enhancer-binding protein α and zinc finger protein 423) [60]. *Tsc1* deletion in Osterix-positive cells led to an mTORC1-dependent downregulation of the Wnt/β-catenin signaling by decreasing the total and active β-catenin protein levels via the non-canonical Notch1-mediated GSK3-independent active β-catenin degradation, which resulted in a decreased mRNA expression of the Wnt/β-catenin target genes.

A further piece in the understanding of the role of the *TSC1* gene in bone metabolism was recently shown by Liu et al. [61]. By using a mouse model with the conditional homozygote deletion of the *Tsc1* gene in osteocytes, the authors showed that the constitutive activation of the mTORC1 signaling in osteocytes enhanced RANKL expression, leading to osteoclast formation, and, at the same time, markedly reduced the expression of sclerostin, a secreted glycoprotein that strongly inhibits osteoblast differentiation and bone formation, via the upregulation of Sirt1, a histone deacetylase repressor of the sclerostin gene expression. As a consequence, the mutated mice manifested osteosclerosis with enhanced bone formation and increased BMD. 

One study investigated, in vivo and in vitro, the effect of the biallelic *Tsc2* depletion in murine osteoblasts, showing that the *Tsc2* deficiency-induced mTORC1 activation caused the accumulation of disorganized bone tissue as a consequence of an excessive number of active osteoblasts, indicating that tuberin is required for normal osteoblast differentiation [62].

Sclerotic bone lesions (SBLs), presumably originating from cells of the neural crest, are a common skeletal manifestation in patients affected by Tuberous Sclerosis (TSC) syndromes. They manifest in about 84% of mutated patients (47.2% with mutated *TSC1* gene and 52.8% with mutated *TSC2* gene), but only in 14% of patients without *TSC1* or *TSC2* mutations [63], suggesting that the inactivation of the hamartin-tuberin complex, and the subsequent hyper-activation of mTOR signaling, may have a direct role in the development of SBLs, as the studies described above also appear to confirm. 

## 4. Conclusions 

Except for pathognomonic skeletal abnormalities related to some of the syndromic inherited endocrine tumors that are commonly included in the diagnosis of the disease and that are normally described and discussed in the clinical cases, the majority of bone and mineral metabolism affections in these congenital disorders have long remained poorly investigated in the clinical practice, and are still largely missing in the medical literature, guidelines and patients’ medical records.

However, despite the fact that these clinical manifestations are less severe than the associated endocrine tumors, they are a cause of morbidity and disability and contribute to reduce the quality of life of affected patients.

- Medical interventions aimed to prevent the development of some of these bone affections, such as osteopenia and osteoporosis, or to early diagnose, control and treat those that cannot be prevented, should become part of the clinical and therapeutic management of hereditary endocrine tumors.
- A more and more detailed knowledge of these bone manifestations, deriving from the routine description of their clinical aspects and biological features by clinicians and from their inclusion in the clinical guidelines of inherited endocrine tumors, as well as from basic research and clinical studies investigating the molecular roles of “cancer genes” in the regulation of bone development and metabolism in physiological and pathological conditions, is a key point for designing better clinical managements of these skeletal affections, and for possible future targeted therapies.
- Since some of the mutated genes responsible for the inherited endocrine tumor phenotypes and the subsequent haploinsufficiency of their encoded proteins, such as parafibromin, APC, PRKAR1A, PTEN, NF1, tuberin and hamartin, were shown to be directly involved in the development of the syndrome-specific skeletal abnormalities, targeting their downstream dysregulated signaling pathways represent potential targets for treatments not only of the tumor manifestations but also of the bone affections.

## Figures and Tables

**Table 1:** Causative genes, main endocrine and non-endocrine clinical manifestations and skeletal features in inherited endocrine tumors.

Disease Name [OMIM Number]	Causative Gene (Chromosomal Location)	Gene Function	Type of Germline Mutations	Main Endocrine and Non-Endocrine Clinical Manifestations	Pathognomonic Skeletal Features	Non-Pathognomonic Bone and Mineral Metabolism Alterations, Reported in Affected Patients
**Non-syndromic inherited endocrine tumors**						
Familial isolated primary hyperparathyroidism (FIHP) [145000]	*CDC73* (1q31.2)	TSG	Heterozygote inactivating mutations of the *CDC73* gene.	Parathyroid hyperplasia, adenomas and carcinoma. Primary hyperparathyroidism only.	Not reported	Hyperparathyroidism-derived bone mass loss
Autosomal dominant familial isolated hyperparathyroidism type 4 (HRPT4) [617343]	*GCM2* (6p24.2)	Oncogene	Heterozygote activating mutations of the *GCM2* gene.	Parathyroid hyperplasia, adenomas and rare cases of carcinoma. Primary hyperparathyroidism only.	Not reported	Hyperparathyroidism-derived bone mass loss (osteopenia) and/or kidney stones.
Familial isolated pituitary adenoma 1, multiple types (FIPA) [102200]	*AIP* (11q13.2)	TSG	Up to 20% of FIPA patients have germline heterozygote inactivating mutations of the *AIP* gene, with a tumor penetrance of 20–23% [2].	Mostly pituitary GH-secreting adenomas, but also pituitary ACTH-secreting, PRL-secreting and TSH-secreting adenomas. Pituitary tumor type heterogeneity among family members.	GH-secreting tumors cause acromegaly (coarse facial features, protruding jaw and enlarged extremities) and gigantism.	Not reported
X-linked acrogigantism (XLAG) [300942]	*GPR101* (Xq26.3)	Oncogene	*De novo* germline microduplication of the *GPR101* gene.	GH-secreting pituitary hyperplasia/adenoma.	Skeletal overgrowth caused by excessive GH	Not reported
Familial adenomatous polyposis type 1 (FAP1) [175100]	*APC* (5q22.2)	TSG	Heterozygote inactivating mutations of the *APC* gene. The great majority of the known germline mutations result in a truncation of the APC protein. Exon 15 represents a “mutation cluster region”.	Hundreds to thousands adenomatous polyps (adenomas) of the colon and rectum. Predisposing factor to carcinoma of the colon and the rectum.	Osteomas (benign, slow growing bony tumors involving the base of the skull and paranasal sinuses and originating in bones characterized by intramembranous ossification) in the Gardner syndrome variant. Dental anomalies, such as supernumerary and impacted teeth [3]	Not reported
**Syndromic inherited endocrine tumors**						
Multiple Endocrine Neoplasia type 1 (MEN1) [131100]	*MEN1* (11q13.1)	TSG	Heterozygote inactivating mutations of the *MEN1* gene (no mutation cluster region). The great majority of the known mutations result in a truncation of the menin protein [4].	Parathyroid four-gland hyperplasia/adenoma, neuroendocrine tumors of the gastro-entero-pancreatic tract, pituitary adenoma (mostly PRL-secreting tumors), bronchial and thymic carcinoids, hyperplasia/adenoma of the adrenal glands.	Not reported	Hyperparathyroidism-derived early onset bone mass loss. Significantly higher prevalence of severe osteopenia and osteoporosis, mainly in women by the age of 35, with respect to control population of the same age [5]. Increased risk of fractures. GH-secreting tumors cause gigantism in children and acromegaly in adults.
Multiple Endocrine Neoplasias type 2A (MEN2A) [171400] and type 2B (MEN2B) [162300]	*RET* (10q11.21)	Oncogene	Heterozygote gain-of-function missense mutations of the *RET* gene, affecting one of the 6 extracellular cysteines of the RET protein (MEN2A) or one of the two intracellular tyrosine-kinase domains (MEN2B).	Medullary thyroid carcinoma, pheochromocytoma and, only in the MEN2A form, parathyroid hyperplasia/adenoma.	Marfanoid habitus (slender, tall and thin body with long limbs), high-arched palate, long and thin face with prognathism, pectus excavatum, equino-varus foot, femoral epiphysiolysis, kyphosis, scoliosis in MEN2B.	Hyperparathyroidism-derived bone mass reduction in MEN2A.
Multiple Endocrine Neoplasia type 4 (MEN4) [610755]	*CDKN1B* (12p13.1)	TSG	Heterozygote inactivating mutations of the *CDKN1B* gene (no mutation cluster region).	Parathyroid multiglandular hyperplasia/adenoma, pancreatic neuroendocrine tumors, pituitary adenomas.	Not reported	Possible hyperparathyroidism-derived bone mass reduction after 45 years of age.
Hyperparathyroidism-jaw tumors syndrome (HPT-JT) [145001]	*CDC73* (1.q31.2)	TSG	Heterozygote inactivating mutations of the *CDC73* gene (no mutation cluster region).	Parathyroid hyperplasia, adenomas and/or carcinoma, ossifying fibroma of the maxilla and/or mandible, renal tumors (cysts, hamartomas and/or renal cell cancer) and uterine fibromas.	Ossifying fibroma of the maxilla and/or mandible.	Parathyroid carcinoma-derived severe hyperparathyroidism and hypercalcemia lead to a high bone resorption, osteopenia/osteoporosis, bone pain, increased risk of fragility fractures, hypercalciuria and nephrolithiasis, and they may give rise to multiple brown tumors (osteitis fibrosa cystica).
Von Hippel-Lindau syndrome (VHL) [193300]	*VHL* (3p25.3)	TSG	Heterozygote inactivating mutations of the *VHL* gene.	Angiomas of the retina, hemangioblastoma of the cerebellum and hemangioma of the spinal cord. Renal cell carcinoma, pheochromocytoma/paraganglioma and pancreatic tumors (usually non-functioning) are reported in some patients.	Not reported	Not reported
Carney complex type 1 (CNC1) [160980]	*PRKAR1A* (17q24.2)	TSG	Heterozygote inactivating mutations of the *PRKAR1A* gene.	Multiple cardiac, endocrine, cutaneous and neural myxomatous tumors. A variety of pigmented lesions of the skin and mucosae (multiple lentigines, ephelides, nevi) in typical sites (conjunctiva, lips, genital mucosa). Large cell calcifying Sertoli cell tumor, psammomatous melanotic schwannomas.	Myxoid bone tumors (osteochondromyxomas), prevalently located in the nasal region and in the diaphysis of radius and tibia.	Not reported
Cowden/PTEN hamartoma tumor syndrome (PHTS) [158350]	*PTEN* (10q23.31)	TSG	Heterozygote inactivating de novo mutations of the *PTEN* gene in 10–44% of PHTS patients, with a 100% penetrance by adulthood [6]	Multiple hamartomas. Macrocephaly, adult Lhermitte-Duclos disease, facial trichilemmomas, acral keratoses, papillomatous papules. Increased risk of developing carcinomas of the breast, the thyroid (follicular or papillary tumors) and the endometrium.	Increased cranial size (macrocephaly), with an occipital frontal circumference ≥ 97th percentile. Skeletal abnormalities such as kyphosis, kyphoscoliosis, pectus excavatum, large hands and feet, syndactyly, hypoplasia of the mandible, the maxilla and the scapulae [7].	Osteosarcoma is an extremely rare presentation of PHTS (only one case has been reported in the English-written literature) [8].
Neurofibromatosis type 1 (NF1) [162200]	*NF1* (17q11.2)	TSG	Heterozygote inactivating mutations of the *NF1* gene.	Multiple benign tumors of the nerves (neurofibromas), peripheral nerve sheath tumors, pigmented skin lesions (Cafe-au-lait spots, ephelides), Lisch nodules in the eye, optic pathway gliomas, fibromatous tumors of the skin.	Characteristic bone deformities have been reported in about 35% of patients [9], including congenital anterolateral bowing of the tibia (characterized by recurrent fracture of the lower leg in early childhood and pseudoarthrosis), dysplasia of the sphenoid wing, congenital severe dystrophic scoliosis, macrocephaly, abnormalities of the rib cage (rib fusion), lytic bone lesions, absence of the patella, syndactyly. Recalcitrant bone healing after fracture (pseudoarthrosis).	Higher incidence of osteopenia/osteoporosis (over 50% of patients showed a significant overall reduction in BMD starting from childhood), compared to the general population of the same age [9]. Alterations of bone mineral metabolism, significant reduction in endochondral bone formation, increased collagen synthesis.
Tuberous sclerosis type 1 (TSC1) [191100] and type 2 (TSC2) [613254]	*TSC1* (9q34.13) *TSC2* (16p13.3)	TSG TSG	Germline mutations account for 50–86% of all cases of tuberous sclerosis complex. TSC1 is caused by heterozygous inactivating mutations in the *TSC1* gene (approximately 10 to 30% of cases). TSC2 is caused by heterozygous inactivating mutations in the *TSC2* gene (about 50% of cases). *TSC2* mutations are associated with a more severe disease.	Multiple hamartomas in multi organ systems (brain, kidneys, skin, heart and lungs). Central nervous system manifestations (epilepsy, seizures, learning difficulties, autism). Renal cysts, angiomyolipomas and renal cell carcinoma. Skin lesions (hypomelanotic macules, facial angiofibromas, confetti lesions, patches of connective tissue nevi). Pulmonary lymphangioleiomyomatosis.	Sclerotic bone lesions (in about 40–60% of cases), including hyperostosis of the inner table of the calvaria and cyst-like osseous lesions (mainly at metacarpal and metatarsal bones, spine, pelvis and calvaria) [10]. Thickening of cranial neural crest-derived bones (frontal bones, mandibles, maxilla).	Osteoblastic changes, excess of periosteal new bone formation.

Footnotes: TSG = tumor suppressor gene; GH = growth hormone; ACTH = adrenocorticotropic hormone; PRL = prolactin; TSH = thyroid stimulating hormone.

## Data Availability

Not applicable.
